# Pilot study of risk factors associated with cardiovascular disease in northern and southern Cameroonians

**DOI:** 10.5830/CVJA-2016-094

**Published:** 2017

**Authors:** Jeanne Durendale Chiadak, Jason Perret, Christine Delporte, Jeanne Durendale Pascual, Hilaire Macaire Womeni, Jules Roger Kuiaté, Pierre Cullus, Christelle Senterre

**Affiliations:** Laboratory of Pathophysiological and Nutritional Biochemistry, Faculty of Medicine, Université Libre de Bruxelles, Brussels, Belgium; Jeanne Durendale; Laboratory of Pathophysiological and Nutritional Biochemistry, Faculty of Medicine, Université Libre de Bruxelles, Brussels, Belgium; Section of Nuclear Medicine and Diagnostic Imaging, Division of Human Health, International Atomic Energy Agency, Vienna, Austria; Section of Nuclear Medicine and Diagnostic Imaging, Division of Human Health, International Atomic Energy Agency, Vienna, Austria; Section of Nuclear Medicine and Diagnostic Imaging, Division of Human Health, International Atomic Energy Agency, Vienna, Austria; Research Centre of Epidemiology, Biostatistics and Clinical Research, School of Public Health, Université Libre de Bruxelles, Belgium

**Keywords:** cardiovascular diseases, risk factor, lifestyle, nutrition, diet, fatty acid

## Abstract

**Aim:**

The aim of the study was to evaluate whether the risk factors for cardiovascular disease (CVD) are similar in the northern and southern regions of Cameroon.

**Methods:**

The participants answered a questionnaire concerning their lifestyle. Anthropometric and blood pressure measurements were evaluated in 192 individuals and biochemical parameters in 50 randomly selected volunteers.

**Results:**

Northerners displayed low alcohol and tobacco consumption, little practice of sport but physically demanding professions, and consumption of soybean, refined palm and other polyunsaturated oils. Southerners consumed alcohol, practiced sport, had intellectually based professions, and consumed crude and refined palm oils. Waist circumference and body mass index were higher in the southerners compared to the northerners. Blood glucose levels, and systolic and diastolic blood pressures were higher among the northerners than the southerners. Among the southerners, there were positive correlations between total cholesterol levels and systolic or diastolic blood pressure, low-density lipoprotein cholesterol and blood glucose levels or diastolic blood pressure, triglyceride levels and systolic blood pressure.

**Conclusion:**

Providing region-adapted, health-related advice for northern and southern Cameroonians would contribute to reducing risk factors for CVD.

## Aim

Cardiovascular disease (CVD) remains the leading cause of death worldwide, accounting for around 17 million deaths per year.^1^ In sub-Saharan Africa, CVD is responsible for 20% of all global deaths,^2^ where in the past, CVD was considered a rare ailment among black African populations. However, the incidence of CVD has recently been rising with the increasing shift to Western lifestyle and habits, notably with an increased sodium and calorie intake. These changes account for the rising prevalence of obesity, the metabolic syndrome and diabetes. Between 1990 and 2020, the increase in mortality rates from CVD is estimated to be 140% in men and 118% in women in sub-Saharan Africa, compared to 53% in men and 31% in women in developed countries.^1^,^3^

Monitoring of CVD is based on the evaluation of non-modifiable risk factors (age, gender, race, family history), behavioural risk factors (physical inactivity, excessive alcohol consumption, smoking, diet high in saturated fat, salt and cholesterol), and modifiable risk factors (obesity, diabetes, hypertension, low- and high-density lipoprotein cholesterol levels).^4^

In developing countries, relationships between malnutrition and CVD have been poorly explored.^5^-^7^ Cameroon is considered a miniature Africa due to its diverse climate (ranging from southern tropical forests to northern savannah and western mountains) and its ethnic diversity (366 ethnic groups and 240 languages).

The various ethnic groups distributed across 10 regions, including rural and urban zones, are characterised by distinct eating habits. The second Cameroonian household survey assessed the average energy intake per capita to be 3 241, 2 721 and 2 887 kcal/day for far north, far south and the whole country, respectively.^8^ Contribution to energy requirements results from three main nutrient uptakes; protein, fat and carbohydrates. The percentage energy from protein per unit of consumption was 11.9% in the far north, 9.4% in the far south and 10.5% overall for Cameroon. The percentage energy from fat per unit of consumption was 19.8% in the far north, 28.8% in the far south and 27.1% overall for Cameroon. Finally, the percentage energy from carbohydrate per unit of consumption was 68.3% in the far north, 61.7% in the far south and 62.4% overall for Cameroon.^8^ The percentage recommended for these three nutrients are 50 to 55% for carbohydrates, 12 to 15% for proteins and 30 to 38% for fat.

In Cameroon, crude palm oil represents 10.6%, cottonseed oil 2.1%, refined palm oil 0.9% and peanut oil 0.4% of the total energy intake.^8^ Changes in the amount and/or type of fatty acids ingested can contribute to the development of many diseases, including obesity and diabetes, and thereby CVD. It appears that fatty acids could be implicated in the pathogenesis of these diseases; either by altering the composition of membrane phospholipids, or by modulating the expression of genes.^9^

Plasma omega-3 fatty acid levels were correlated to the eating habits in the three European countries investigated.^10^ The consumption of palm oil, which is rich in saturated fatty acids, is a well-recognised risk factor for cardiovascular and metabolic diseases; palm oil induces a larger increase in plasma concentrations of total cholesterol and low-density lipoprotein cholesterol.^11^ It has been suggested that a 20% increase in the tax on palm oil in India would reduce mortality rates from CVD by 1.3% and reduce total cholesterol levels by 0.08 mmol/l without substitution of palm oil with other oils.^12^ Furthermore, if palm oil could be substituted by an oil rich in polyunsaturated fatty acids, total cholesterol levels would then be reduced by 0.10 mmol/l. A reduction of 0.009 mmol/l in cholesterol level has been considered clinically significant, with a noticeably beneficial impact on health.^13^

In this context, the impact of dietary lipids on the development of CVD represents the focus of current health concerns, not only in economically developed countries but also in developing countries. While eating behaviours differ between the north and south of Cameroon, the energy intake remains comparable between the two regions, 3 241 kcal/day in the north and 2 721 kcal/day in the south.^8^ The aim of this study was therefore to evaluate whether the risk factors for CVD are similar in north and south Cameroonians displaying different food habits (regarding fat intake) but characterised by equivalent average energy intake.

## Methods

This study was performed on 192 volunteers, composed of 101 women and 91 men aged 35 to 65 years. They were distributed between 89 volunteers from the north (45 men and 44 women) and 103 volunteers from the south (46 men and 57 women). The volunteers answered a questionnaire concerning their lifestyle. In addition, their anthropometrics and biochemical parameters were measured.

This study (P2013/164) was approved by the ethics committee of the Université Libre de Bruxelles, Erasme University Hospital and was conducted in compliance with the Declaration of Helsinki. All volunteers gave written informed consent before participation.

The sample size was calculated using the following standard formula: [n = t^2^ × p × (1–p)/m^2^] where n is the minimum sample size to obtain significant results for an event and a fixed level of risk; t is the confidence level (typical value of the confidence level of 95% is 1.96); p is the estimated prevalence of CVD, based on the literature (14% mortality rate due to CVD in Cameroon14) and m is the margin of error (usually set at 5%).

Due to logistical and financial reasons, the biochemical parameters were measured in 50 randomly selected volunteers (25 north, 25 south, with equal numbers of men and women from the different cities). Anthropometric and blood pressure measurements were performed on all 192 volunteers.

All volunteers were living in the urban zone from different cities of the south (S): Douala, Yaoundé, Bafang and Dschang; and the north (N): Garoua and Ngaoudéré. All volunteers belonged to the working and middle social classes. The volunteers were randomly recruited in their home or work place.

Basic information was collected and included the following: gender: female (F) and male (M); age: volunteers were grouped by age: 35–50 and 51–65 years; tobacco consumption: defined as never smoked (no); former smoker or smoker (yes); alcohol consumption: defined as no consumption (none); consumption once or twice a week (occasionally); consumption more than twice a week (regularly); physical activity (sport and professional) was defined as sport: never practice (no); practice intense or moderate activity once or more than once a week (yes); for profession, domestic workers, farmers and traders were considered physically active, whereas office workers were considered intellectual; oil consumption: the oils mostly used for cooking were recorded.

Various anthropometric parameters were collected for all volunteers. Height was collected from the ID card and weight was measured using an electronic scale. Body mass index (BMI) was calculated using the formula: weight (kg)/height^2^ (m) and BMI categories were defined as underweight with BMI ≤ 18.5 kg/m^2^; normal was BMI 18.5–24.9 kg/m^2^; overweight was BMI 25–29.9 kg/m^2^; and obese was BMI ≥ 30 kg/m^2^.^15^ Waist circumference (WC) was measured with a tape measure at the navel while standing. The recommended WC thresholds for assessing abdominal obesity for sub-Saharan African was: women ≥ 80 cm; men ≥ 94 cm.^15^

Systolic and diastolic blood pressure measurements were performed on all volunteers in a seated position at rest and monitored for at least five minutes using an electronic medical wrist blood pressure monitor (Omron M2 Basic; Omron Electronics SA, Knokke-Heist, Belgium). Two measurements were performed on each subject and the mean value was used. Blood pressure ranges were for systolic blood pressure (SBP): normal < 140 mmHg, and high ≥ 140 mmHg; for diastolic blood pressure (DBP): normal < 90 mmHg, and high ≥ 90 mmHg.^16^

Biochemical parameters such as blood glucose (BG), total cholesterol (TC), high-density lipoprotein cholesterol (HDL-C) and triglycerides (TG), were measured in 50 randomly selected volunteers, using an equal number of men and women from different cities, who were advised to fast prior to and up to the time of measurement (not having eaten and/or drunk during the last 12 or eight hours, respectively).

Disposable test strips were used with a Cardio Chek Analyser (Polymer Technology systems Inc, Indianapolis, USA). Low-density lipoprotein cholesterol (LDL-C) was calculated using Fridewald’s formula: LDL-C = TC – (HDL-C + TG/5).

Blood glucose values were defined as normal < 100 mg/dl (5.55 mmol/l); limit 100–125 mg/dl (5.55–6.94 mmol/l); diabetic > 125 mg/dl (> 6.94 mmol/l).

TC ranges were defined for women as normal: 155–255 mg/dl (4.01–6.6 mmol/l); hypocholesterolaemia: < 155 mg/dl (< 4.01 mmol/l) and hypercholesterolaemia: 255 mg/dl (> 6.6 mmol/l). TC ranges were defined for men as normal: 130–250 mg/dl (3.37–6.48 mmol/l); hypocholesterolaemia: < 130 mg/dl (< 3.37 mmol/l) and hypercholesterolaemia: > 250 mg/dl (> 6.48 mmol/l).

HDL-C ranges were defined for women as normal: 50–92 mg/dl (1.3–2.38 mmol/l); hypocholesterolaemia: < 50 mg/dl (< 1.3 mmol/l), and hypercholesterolaemia: > 92 mg/dl (> 2.38 mmol/l). HDL-C ranges were defined for men as normal: 37–65 mg/dl (0.96–1.68 mmol/l); hypocholesterolaemia: < 37 mg/dl (0.96 mmol/l) and hypercholesterolaemia: > 65 mg/dl (1.68 mmol/l).

LDL-C ranges were defined for women as normal: 100–150 mg/dl (2.59–3.89 mmol/l), hypocholesterolaemia: < 100 mg/ dl (< 2.59 mmol/l) and hypercholesterolaemia: > 150 mg/dl (> 3.89 mmol/l). LDL-C ranges were defined for men as normal: 110–160 mg/dl (2.85–4.14 mmol/l), hypocholesterolaemia: < 110 mg/dl (< 2.85 mmol/l) and hypercholesterolaemia: > 160 mg/dl (> 4.14 mmol/l).

Triglyceride ranges were defined for women as normal: 35–140 mg/dl (0.4–1.58 mmol/l), hypotriglyceridaemia: < 35 mg/ dl (< 0.4 mmol/l) and hypertriglyceridaemia: > 140 mg/dl (> 1.58 mmol/l). Triglyceride ranges were defined for men as normal: 45–175 mg/dl (0.51–1.98 mmol/l), hypotriglyceridaemia: < 45 mg/dl (> 0.51 mmol/l) and hypertriglyceridaemia: > 175 mg/dl (> 1.98 mmol/l).^15^

## Statistical analysis

All statistical tests were performed using SPSS software (version 22.0.0.0; IBM Corp). For quantitative variables, data are presented as mean or median ± standard error of means (SEM). The Student’s t-test for independent samples or Wilcoxon– Mann–Whitney test were used to compare the means or medians of two groups, while one-way ANOVA or the Kruskal–Wallis test were used to compare the means or medians of more than two groups. For qualitative variables, data are presented in tables using numbers and percentages, and chi-squared or Fisher’s exact tests were used to compare characteristics between the groups. Spearman’s correlations were used to test possible correlations between two parameters. The results were considered statistically significant for p < 0.05.

## Results

The study population was distributed equally between men and women in the two regions, north and south. The distribution according to age group showed a significant difference (p < 0.001) between the two regions.

Populations from the south consumed more alcohol than those in the north, who drank little or nothing (p < 0.001). The tobacco consumption was zero in the north and in the south very low (p < 0.03).

The intense or moderate practice of sport was very low in the north compared to the south (p < 0.001). However, northerners had physical rather than intellectual professions compared to people of the south (p < 0.001).

The percentage consumption of polyunsaturated oils was higher among the northerners compared to southerners. The majority of the southern population consumed both crude and refined palm oils, while a small portion of the southern people consumed only crude palm oil. In the north, people consumed mainly soybean oil and refined palm oil (Table 1).

**Table 1:** Characteristics of the study population

**	*Population*			*Percentage (%)*		**
*Characteristics*	*Total number*	*North (n = 89)*	*South (n = 103)*	*North*	*South*	*p-value*
Gender						
Female	101	44	57	49	55	0.23
Male	91	45	46	51	45	
Age (years)						
35–51	107	64	43	72	42	< 0.001
51–65	85	25	60	28	58	
Alcohol use						
None	96	88	8	99	8	< 0.001
Occasionally	88	0	88	0	85	
Regularly	8	1	7	1	7	
Tobacco use						
Yes	9	0	9	0	9	0.03
No	183	89	94	100	91	
Sport						
Yes	35	4	31	5	30	< 0.001
No	157	85	72	95	70	
Profession						
Physical	146	83	63	93	61	< 0.001
Intellectual	46	6	40	7	39	
Oils consumed
Crude palm oil	30	0	30	0	29	< 0.001
Crude and refined palm oils	71	0	71	0	69	
Soybean oil and refined palm	75	75	0	84	0	
Other polyunsaturated oils	16	14	2	16	2	

p-value of chi-squared or Fischer’s exact test for the comparison of percentages between the two regions.

WC and BMI were higher among southerners than northerners (p = 0.01 and 0.04, respectively). However, the percentage of obese population, determined by measuring either the WC (north 61%, south 69%) or BMI (north 21%, south 21%), was statistically comparable between the two regions.

The average and range of TC, HDL-C, LDL-C and TG levels were not significantly different according to region. By contrast, BG levels, SBP and DBP were higher in the northern populations than the south (p = 0.024, p < 0.001, respectively). The percentages of individuals with diabetes, 10% in the north and 2% in the south, were not statistically different. There was however an over-representation of hypertension (SBP and DBP) in the north compared to the south (p < 0.001) (Table 2).

**Table 2:** Anthropometric and biochemical parameters according to region

*Parameters*	*North*	*South*	*p-value*
WC (cm) (n =192)			
Mean ± SEM	88 ± 10	92 ± 10	0.01
Normal, n (%)	35 (39)	32 (31)	0.32
Obese, n (%)	54 (61)	71 (69)	
BMI (kg/m^2^) (n =192)			
Mean ± SEM	26 ± 4	28 ± 5	0.04
Normal, n (%)	40 (45)	31 (30)	0.10
Overweight, n (%)	30 (34)	50 (49)	
Obese, n (%)	19 (21)	22 (21)	
BG (mg/dl) (n =192)			
Mean ± SEM	105 ± 51	92 ± 18	0.02
[mmol/l]	[5.83 ± 2.83]	[5.11 ± 1.00]	
Normal, n (%)	67 (75)	81 (79)	0.31
Limit, n (%)	13 (15)	20 (19)	
Diabetes, n (%)	9 (10)	2 (2)	
TC (mg/dl) (n = 50)			
Mean ± SEM	196 ± 56	193 ± 44	0.83
[mmol/l]	[5.08 ± 1.45]	[5.00 ± 1.14]	
Normal, n (%)	18 (80)	18 (72)	1
Hypocholesterolaemia, n (%)	3 (12)	4 (16)	
Hypercholesterolaemia, n (%)	4 (16)	4 (16)	
HDL-C (mg/dl) (n = 50)			
Mean ± SEM	60 ± 16	61 ± 16	0.89
[mmol/l]	[1.55 ± 0.41]	[1.58 ± 0.41]	
Normal, n (%)	15 (60)	17 (68)	0.71
Hypocholesterolaemia, n (%)	6 (24)	3 (12)	
Hypercholesterolaemia, n (%)	4 (16)	5 (20)	
LDL-C (mg/dl) (n = 50)			
Mean ± SEM	111 ± 48	109 ± 38	0.83
[mmol/l]	[2.87 ± 1.24]	[2.82 ± 0.98]	
Normal, n (%)	7 (28)	8 (32)	1
Hypocholesterolaemia, n (%)	15 (60)	14 (56)	
Hypercholesterolaemia, n (%)	3 (12)	3 (12)	
TG (mg/dl) (n = 50)			
Mean ± SEM	121 ± 91	116 ± 56	0.81
[mmol/l]	[1.37 ± 1.03]	[1.31 ± 0.63]	
Normal, n (%)	21 (84)	18 (72)	0.49
Hypocholesterolaemia, n (%)	0 (0)	0 (0)	
Hypercholesterolaemia, n (%)	4 (15)	7 (28)	
SBP (mmHg) (n =192)	146 ± 25	132 ± 20	< 0.001
Mean ± SEM	42 (47)	76 (74)	< 0.001
Hypertensive, n (%)	47 (53)	27 (26)	
DBP (mmHg) (n = 192)			
Mean ± SEM	96 ± 18	84 ± 15	< 0.001
Normal, n (%)	26 (29)	68 (66)	< 0.001
Hypertensive, n (%)	63 (71)	35 (34)	

p-value for the comparison of means by Student’s t-test for independent samples or Wilcoxon–Mann–Whitney test and chi-squared or Fischer’s exact test for the comparison of the percentages in the two regions.

WC, waist circumference; BMI, body mass index; BG, blood glucose; TC, total cholesterol; HDL-C, high-density lipoprotein cholesterol; LDL-C, low-density lipoprotein cholesterol; TG, triglycerides; SBP, systolic blood pressure; DBP, diastolic blood pressure.

In men from the two regions, there was no statistically significant difference between WC and BMI. By contrast, women from the south had a WC and BMI statistically higher than northern women (p = 0.001 and 0.005, respectively). The percentage of obesity was statistically higher among northern than southern men, based on BMI (p = 0.03) but not WC. By contrast, the percentage of obese women was higher in the south compared to the north, based on WC (p < 0.001) but not BMI.

BG, TC, HDL-C, LDL-C and TG levels were not significantly different between women and men in the two regions. Both SBP and DBP were higher in men from the north than from the south (p < 0.001), whereas those parameters were comparable in women from the two regions.

Northern men had a higher incidence of diabetes (16%) than men from the south (4%) (p = 0.027). Similarly, a greater percentage of men from the north had a SBP and DBP higher than that of men from the south (p < 0.001). The incidence of hypertension was higher among women from the north than from the south (p = 0.01) (Table 3).

**Table 3:** Anthropometric and biochemical parameters among subjects of the same gender from the two regions

*Parameters*	*NM*	*SM*	*p-value*	*NW*	*SW*	*p-value*
WC (cm) (n = 192)						
Mean ± SEM	89 ± 9	88 ± 9	1	87 ± 10	95 ± 11	0.001
Normal, n (%)	27 (60)	32 (70)	0.19	8 (18)	0 (0)	< 0.001
Obese, n (%)	18 (40)	14 (30)		36 (82)	57 (100)	
BMI (kg/m^2^) (n = 192)			
Mean ±SEM	25 ± 4	24 ± 2	1	27 ± 5	30 ± 6	0.005
Normal, n (%)	23 (52)	24 (52)	0.68	17 (39)	7 (12)	0.01
Overweight, n (%)	12 (27)	19 (41)	0.23	18 (41)	31 (54)	0.27
Obese, n (%)	9 (21)	3 (7)	0.03	9 (20)	19 (34)	0.13
BG (mg/dl) (n = 192)						
Mean ± SEM	112 ± 61	91 ± 20	0.09	98 ± 38	92 ± 17	1
[mmol/l]	[6.22 ± 3.39]	[5.05 ± 1.11]	0.07	[5.44 ± 2.11]	[5.11 ± 0.94]	0.86
Normal, n (%)	34 (75)	35 (76)		33 (75)	42 (74)	
Limit, n (%)	4 (9)	9 (20)		9 (20)	11 (19)	
Diabetes, n (%)	7 (16)	2 (4)	0.02	2 (4)	4 (7)	
TC (mg/dl) (n = 50)						
Mean ± SEM	214 ± 71	196 ± 48	0.87	181 ± 34	191 ± 41	0.89
[mmol/l]	[5.54 ± 1.84]	[5.08 ± 1.24]	1	[4.69 ± 0.88]	[4.95 ± 1.06]	0.64
Normal, n (%)	9 (75)	9 (69)		9 (69)	9 (75)	
Hypocholesterolaemia, n (%)	0 (0)	1 (7)		4 (30)	2 (16)	
Hypercholesterolaemia, n (%)	3 (25)	3 (23)		0 (0)	1 (8)	
HDL-C (mg/dl) (n = 50)						
Mean ± SEM	58 ± 15	58 ± 17	1	62 ± 18	65 ± 14	1
[mmol/l]	[1.50 ± 0.39]	[1.50 ± 0.44]	1	[1.61 ± 0.47]	[1.68 ± 0.36]	0.64
Normal, n (%)	6 (50)	7 (53)		9 (69)	10 (83)	
Hypocholesterolaemia, n (%)	2 (16)	1 (7)		4 (31)	2 (16)	
Hypercholesterolaemia, n (%)	4 (33)	5 (38)		0 (0)	0 (0)	
LDL-C (mg/dl) (n = 50)						
Mean ± SEM	127 ± 54	112 ± 42	1	96 ± 38	105 ± 35	1
[mmol/l]	[3.29 ± 1.40]	[2.90 ± 1.09]	1	[2.49 ± 0.98]	[2.72 ± 0.91]	0.83
Normal, n (%)	3 (25)	3 (23)		4 (30)	5 (41)	
Hypocholesterolaemia, n (%)	7 (58)	8 (61)		8 (61)	6 (50)	
Hypercholesterolaemia, n (%)	2 (17)	2 (15)		1 (7)	1 (8)	
TG (mg/dl) (n = 50)						
Mean ± SEM	137 ± 124	125 ± 67	1	106 ± 45	106 ± 42	1
[mmol/l]	[1.55 ± 1.4]	[1.41 ± 0.76]	0.64	[1.20 ± 0.51]	[1.20 ± 0.47]	0.64
Normal, n (%)	10 (83)	9 (60)		11 (84)	9 (75)	
Hypocholesterolaemia, n (%)	0 (0)	0 (0)		0 (0)	0 (0)	
Hypercholesterolaemia, n (%)	2 (16)	4 (30)		2 (15)	3 (25)	
SBP (mmHg) (n = 192)						
Mean ± SEM	152 ± 27	129 ± 19	< 0.001	139 ± 22	134 ± 22	1
Normal n (%)	16 (35)	36 (78)	< 0.001	26 (59)	40 (71)	0.28
Hypertensive, n (%)	29 (64)	10 (22)		18 (40)	17 (29)	
DBP (mmHg) (n = 192)						
Mean ± SEM	98 ± 18	80 ± 14	< 0.001	93 ± 17	87 ± 15	0.32
Normal, n (%)	11 (34)	32 (70)	< 0.001	15 (24)	36 (63)	0.01
Hypertensive, n (%)	34 (75)	14 (30)		29 (66)	21 (37)	

p-value for comparison of means by one-way ANOVA or Kruskal–Wallis test and the chisquared test or Fischer’s exact test for the comparison of percentages between groups.

NM, northern men; NW, northern women; SM, southern men; SW, southern women; WC, waist circumference; BMI, body mass index; BG, blood glucose; TC, total cholesterol; HDL-C, high-density lipoprotein cholesterol; LDL-C, low-density lipoprotein cholesterol;TG, triglycerides; SBP, systolic blood pressure; DBP, diastolic blood pressure.

The characteristics of the populations showed that there was a significant difference in age between the volunteers from the north and south. Taking this into consideration, anthropometric and biochemical parameters among subjects from the two age groups were statistically compared. The results showed no significant difference between the two age groups for WC, BG, SBP and DBP. However, BMI was higher among people in the 35–51-year age group compared to people in the 51–65-year age group (p = 0.01). The percentage of obese people and people with normal SBP tended to be higher among people in the 35–51-year age group compared to people in the 51–65-year age group (16 vs 8%; 37 vs 25%, respectively), but these were not statistically significant (p = 0.08; p = 0.05, respectively) (Table 4).

**Table 4:** Anthropometric and biochemical parameters among subjects of two age groups

*Parameters*	*35–50 years*	*51–65 years*	*p-value*
WC (cm) (n = 192)			
Mean ± SEM	91.48 ± 1.77	91.01 ± 1.92	0.86
Normal, n (%)	37 (19)	69 (36)	0.50
Obese, n (%)	37 (19)	49 (26)	
BMI (kg/m^2^) (n = 192)			
Mean ± SEM	29.42 ± 0.86	26.00 ± 0.93	0.01
Normal, n (%)	37 (19)	33 (17)	0.08
Overweight, n (%)	38 (20)	39 (20)	
Obese, n (%)	31 (16)	14 (8)	
BG (mg/dl) (n = 192)			
Mean ± SEM	92.46 ± 11.63	117.48 ± 12.60	0.15
[mmol/l]	[5.13 ± 0.65]	[6.52 ± 0.70]	
Normal, n (%)	78 (41)	65 (34)	0.42
Limit, n (%)	22 (11)	12 (6)	
Diabetes, n (%)	6 (3)	9 (5)	
SBP (mmHg) (n = 192)			
Mean ± SEM	115.61 ± 8.80	135.17 ± 9.53	0.14
Normal, n (%)	71 (37)	47 (25)	0.05
Hypertensive, n (%)	35 (18)	39 (20)	
DBP (mmHg) (n = 192)			
Mean ± SEM	88.65 ± 2.44	94.14 ± 2.65	0.13
Normal, n (%)	53 (28)	41 (21)	0.43
Hypertensive, n (%)	53 (28)	45 (23)	

p-value for the comparison of means by Student’s t-test for independent samples or Wilcoxon–Mann–Whitney test and chi-squared test for the comparison of percentages between the two age groups.

WC, waist circumference; BMI, body mass index; BG, blood glucose; SBP, systolic blood pressure; DBP, diastolic blood pressure.

In the north, there was a positive correlation between TC and TG levels (r = 0.61, p = 0.001); SBP and DBP (r = 0.82, p < 0.001); SBP and WC (r = 0.30, p = 0.004); SBP and BMI (r = 0.24, p = 0.03) and DBP and WC (r = 0.26, p = 0.01). There was a negative correlation between HDL-C and TG levels (r = –0.45, p = 0.02) (Table 5). In the south there was a positive correlation between TC and TG levels (r = 0.48, p = 0.02); TC and SBP or DBP (r = 0.44, p = 0.03; r = 0.54, p = 0.006, respectively); LDL-C and BG (r = 0.43, p = 0.03); LDL-C and DBP (r = 0.49, p = 0.01) and TG and SBP (r = 0.54, p = 0.006) (Table 6).

**Table 5:** Correlations between parameters in the northerners

**	**	*BG*	*TC*	*HDL-C*	*TG*	*LDL-C*	*SBP*	*DBP*	*WC*	*BMI*
BG	CC	1								
	Sig									
	n	89								
TC	CC	0.13	1							
	Sig	0.54								
	n	25	25							
HDL-C	CC	0.17	-0.03	1						
	Sig	0.41	0.87							
	n	25	25	25							
TG	CC	0.23	0.61	–0.45	1					
	Sig	0.27	0.001	0.02						
	n	25	25	25	25					
LDL-C	CC	0.004	0.95	–0.21	0.49	1				
	Sig	0.98	< 0.001	0.32	0.01					
	n	25	25	25	25	25				
SBP	CC	0.09	–0.20	0.07	–0.18	–0.19	1			
	Sig	0.39	0.32	0.75	0.39	0.36				
	n	89	25	25	25	25	89			
DBP	CC	0.14	–0.16	0.10	0.03	–0.24	0.82	1		
	Sig	0.21	0.44	0.64	0.88	0.26	< 0.001			
	n	89	25	25	25	25	89	89		
WC	CC	0.02	–0.06	0.36	–0.38	–0.05	0.30	0.26	1	
	Sig	0.87	0.77	0.08	0.06	0.81	0.004	0.01		
	n	89	25	25	25	25	89	89	89	
BMI	CC	0.01	0.02	0.29	–0.11	–0.04	0.24	0.19	0.72	1
	Sig	0.93	0.92	0.15	0.61	0.86	0.03	0.07	< 0.001	
	n	89	25	25	25	25	89	89	89	89

CC, correlation coefficient; n, sample size; Sig, p-value; WC, waist circumference; BMI, body mass index; BG, blood glucose; TC, total cholesterol; HDL-C, high-density lipoprotein cholesterol; LDL-C, low-density lipoprotein cholesterol; TG, triglycerides; SBP, systolic blood pressure; DBP, diastolic blood pressure.

**Table 6:** Correlations between parameters in the southerners

**	**	*BG*	*TC*	*HDL-C*	*TG*	*LDL-C*	*SBP*	*DBP*	*WC*	*BMI*
BG	CC	1								
	Sig									
	n	103								
TC	CC	0.36	1							
	Sig	0.07								
	n	25	25							
HDL-C	CC	–0.03	0.25	1						
	Sig	0.88	0.23							
	n	25	25	25						
TG	CC	–0.01	0.48	0.013	1					
	Sig	0.95	0.02	0.951						
	n	25	25	25	25					
LDL-C	CC	0.43	0.89	–0.14	0.25	1				
	Sig	0.03	< 0.001	0.51	0.23					
	n	25	25	25	25	25				
SBP	CC	–0.18	0.44	0.29	0.54	0.22	1			
	Sig	0.08	0.03	0.16	0.006	0.28				
	n	103	25	25	25	25	103			
DBP	CC	–0.02	0.54	0.02	0.39	0.49	0.71	1		
	Sig	0.85	0.006	0.92	0.06	0.01	< 0.001			
	n	103	25	25	25	25	103	103		
WC	Cc	0.19	0.03	0.03	–0.37	0.13	0.12	0.13	1	
	Sig	0.07	0.88	0.90	0.07	0.54	0.25	0.21		
	n	103	25	25	25	25	103	103	103	
BMI	CC	0.13	–0.09	0.03	–0.21	0.04	0.15	0.16	0.80	1
	Sig	0.22	0.68	0.90	0.10	0.86	0.16	0.12	< 0.001	
	n	103	25	25	25	25	103	103	103	103

CC, correlation coefficient; n, sample size; Sig, p-value; WC, waist circumference; BMI, body mass index; BG, blood glucose; TC, total cholesterol; HDL-C, high-density lipoprotein cholesterol; LDL-C, low-density lipoprotein cholesterol; TG, triglycerides; SBP, systolic blood pressure; DBP, diastolic blood pressure.

## Discussion

To the best of our knowledge, CVD risk factors in northern and southern Cameroonians have not been studied before. Average WC and BMI from the studied population were significantly higher in the south than the north. However, the prevalence of obesity, determined by either BMI (± 21%) or WC (± 65%), was comparable between northern and southern Cameroonians, but based on BMI, higher than that in the entire Cameroonian population (± 10%).^14^ In addition, our data are in agreement with a study performed on the urban labour force from Douala (south) showing an obesity prevalence of 23%, based on BMI.^17^

The range limits used to establish each category (normal, overweight and obese) could explain the lack of difference in obesity prevalence between southerners and northerners, despite differences in average BMI and WC values. Based on BMI measurements, southern women displayed higher prevalence of obesity compared to northern women (34 vs 20%). Obesity prevalence determined in southern women (34%) was close to the values determined in the women’s labour force from Douala (36%, based on BMI),^17^ and similar to the prevalence of obesity among urban southern women (33%)18 and rural southern women (33%).^19^

Based on WC measures, in our study, the prevalence of obesity in southern women was 100%, versus 82% for northern women. Our data are higher than that obtained in urban southern women (43%), based on WC.^17^ Based on BMI, our data showed that the prevalence of obesity in northern men was 21%, versus 7% for southern men, which were higher and lower, respectively, than that from the men’s labour force from Douala (18%, based on BMI).^17^

Based on WC measures, in our study, northern men displayed higher prevalence of obesity compared to southern men (40 vs 30%). These values were higher or lower than the obesity prevalence determined for the men’s labour force from Douala (32%, based on WC).^17^ Recruitment methods, population sizes and/or cut-off values for determination of obesity prevalence may have accounted for these differences.

Strong and positive correlations existed between SBP and WC, SBP and BMI, and DBP and WC among the northerners. In the southern rural zone of Cameroon, obesity in women was not correlated with blood pressure and blood glucose values due to their farming activities, which can be equated with permanent physical exercise.^19^ Increased DBP and BMI in the north may have been due to a lack of physical activity (95%), which appeared to be much higher among northerners than among southerners (70%) or the overall Cameroonian population (39%).^14^

Higher WC and BMI values observed among southerners could have been related to their food habits and/or lifestyle, despite comparable total energy intakes observed in south and north Cameroon.^8^ Southerners were characterised by a significantly higher fat consumption than northerners.^8^ In addition, our study showed that crude and refined palm oils rich in saturated fatty acids (45–55%) and mono-unsaturated fatty acids (38–45%)^20^ were the oils mostly used by southerners. A recent report has shown that the replacement of polyunsaturated fatty acid with saturated fatty acid during a hypercaloric state was accompanied by modest weight gain as well as increased markers of endothelial dysfunction and insulin resistance in healthy, normal-weight individuals.^21^

The higher prevalence of obesity among southerners could also have been related to the high percentage of individuals consuming alcohol occasionally or regularly (92%), compared to the low percentage (1%) of northerners consuming alcohol, for cultural reasons. Our data corroborate a previous study performed on Cameroonians from urban workplaces, showing that the prevalence of obesity was positively correlated with excessive alcohol consumption.^17^

Among southerners, strong positive correlations existed between lipid parameters and other cardiovascular risk factors studied (hypertension and diabetes). Palm oil, rich in saturated fatty acids and consumed by southerners, is a well-recognised risk factor for CVD and metabolic diseases, as it induces a larger increase in plasma concentrations of TC and LDL-C.^11^ According to Keys, food composition, not only rich in cholesterol but also in fatty acids, controlled plasma TC concentrations.^22^ Brady et al. showed that consumption of omega-6 poly-unsaturated fatty acids is likely to contribute to insulin resistance.^23^ Diets with a high omega-3/omega-6 fatty acid ratio improve the plasma lipid profile and are known to reduce obesity and improve insulin resistance.^24^ While fish and shellfish are the main sources of omega-3 fatty acid,^25^ the energy intake from fish in Cameroon represents only 0.4% of the total energy intake.^8^

BG, SBP and DBP were significantly higher among northerners than southerners, with northerners being characterised by high carbohydrate consumption compared to southerners.^8^ Consumption of food with a high glycaemic index may account for increased BG levels and blood pressure among northerners. Similarly, consumption of high glycaemic index foods in Japan increased the risk of developing type 2 diabetes.^26^ In our study, the prevalence of diabetes in northern and southern Cameroonians was similar or lower, respectively, compared to the world global prevalence of diabetes estimated in 2004 at 9% among adults aged 18 years and over.^27^ More than 80% of deaths related to diabetes occur in low- and middle-income countries.^28^ It has been shown that a healthy diet, regular physical activity, maintenance of a normal weight and stopping smoking can prevent or delay the onset of type 2 diabetes.^29^ Providing adapted health-related advice to northern and southern Cameroonians would certainly contribute to reducing risk factors for CVD.

Age has been considered a non-modifiable risk factor for the onset of CVD.^4^ However, the CVD risk factors monitored in our study did not increase with age. On the contrary, we observed that BMI was higher in the 35–51-year age group, compared to people in the 51–65-year age group; this could have been linked to a modification of lifestyle. In a recent report, the increase in mortality rate from CVD between 1990 and 2020 is estimated to be 140% in men and 118% in women in sub-Saharan Africa compared to 53% in men and 31% in women in developed countries. This is probably due to a shift to a Western lifestyle and an increased intake of calories in sub-Saharan Africa.^1^,^3^

## Conclusion

The prevalence of obesity was higher in southern Cameroonians (especially women) compared to northern Cameroonians. Lipid parameters (TC, LDL-C and TG) were positively correlated with cardiovascular risk factors such as BG levels, SBP and DBP among southern Cameroonians. The prevalence of diabetes and high blood pressure was higher among northern than southern Cameroonians, despite the lack of correlation between lipid parameters and cardiovascular risk factors assessed among northern Cameroonians.

The main objective of this pilot study was to attract the attention of national health authorities and medical communities to the impact of dietary habits on the increasing prevalence of cardiovascular risk factors by comparison of the two main regions of Cameroon. Hopefully, the risk factors uncovered by this study will encourage public health authorities to promote (financially and logistically) a widespread multicentre study, allowing healthcare personnel to assess the looming health issues and to eventually provide public health recommendations. Furthermore, additional studies aimed at assessing food consumption and lifestyle factors in these two populations would increase our understanding of cardiovascular risk factors in Cameroon.
